# Metabolic Control of Dendritic Cell Activation and Function: Recent Advances and Clinical Implications

**DOI:** 10.3389/fimmu.2014.00203

**Published:** 2014-05-08

**Authors:** Bart Everts, Edward J. Pearce

**Affiliations:** ^1^Department of Pathology and Immunology, Washington University School of Medicine, St. Louis, MO, USA; ^2^Department of Parasitology, Leiden University Medical Center, Leiden, Netherlands

**Keywords:** metabolism, oxidative phosphorylation, mitochondria, glycolysis, TLR signaling, immunogenic dendritic cells, tolerogenic dendritic cells, immunotherapy

## Abstract

Dendritic cells (DCs) are key regulators of both immunity and tolerance by controlling activation and polarization of effector T helper cell and regulatory T cell responses. Therefore, there is a major focus on developing approaches to manipulate DC function for immunotherapy. It is well known that changes in cellular activation are coupled to profound changes in cellular metabolism. Over the past decade there is a growing appreciation that these metabolic changes also underlie the capacity of immune cells to perform particular functions. This has led to the concept that the manipulation of cellular metabolism can be used to shape innate and adaptive immune responses. While most of our understanding in this area has been gained from studies with T cells and macrophages, evidence is emerging that the activation and function of DCs are also dictated by the type of metabolism these cells commit to. We here discuss these new insights and explore whether targeting of metabolic pathways in DCs could hold promise as a novel approach to manipulate the functional properties of DCs for clinical purposes.

## Introduction

Dendritic cells (DCs) play a crucial role in the development of adaptive immune responses during infections and inflammatory diseases, as well as in the regulation of immune homeostasis during steady state, by governing the activation and maintenance of T cell responses. In response to many viral and bacterial infections, DCs promote the generation of effector CD4^+^ T helper 1 (Th1) and CD8^+^ T cell-dominated immune responses, while fungal and parasitic worm infections are predominantly associated with Th17 and Th2 responses, respectively. In addition to these effector responses, DCs can be instructed to become tolerogenic and promote regulatory T cells (Tregs), which regulate effector T cell responses, a process that is crucial for maintenance of immune homeostasis and control of autoimmune disorders and allergies. Because of the powerful immunoregulatory functions of DCs, there has been great interest in delineating the cellular processes that control the different properties of these cells, to ultimately identify ways to manipulate the function of DCs for the rational design of DC-based immune-interventions.

It has long been appreciated, especially in the cancer field, that changes in cellular activation coincide with, and are underpinned by, alterations in cellular metabolic state (1, 2). Importantly, over the last couple of years it is becoming increasingly clear that immune cell activation is also coupled to profound changes in cellular metabolism and that their fate and function are metabolically regulated (3). This has led to the idea that manipulation of cellular metabolism of immune cells can be used to shape innate and adaptive immune responses to our advantage. While most of our understanding in this area has been gained from studies with T cells (4–6) and macrophages (7, 8), evidence is emerging that metabolic processes also control the activation and immune-priming functions of DCs. In the current review, we will discuss these recent findings and explore whether targeting of metabolic pathways in DCs could hold promise as a novel approach to manipulate their functional properties for DC-based immunotherapy.

## Role of Cellular Metabolism in DC function

Under non-inflammatory conditions, most DCs reside in peripheral tissues where they exist in a resting immature state. In this quiescent state, DCs are poorly immunogenic. However, upon triggering of a set of germline-encoded pattern recognition receptors, including Toll-like receptors (TLRs) by pathogen-derived products or inflammatory stimuli, DCs undergo a well-characterized process of cellular activation, termed DC maturation, which renders them highly immunogenic. This process involves an increase in capturing and processing of antigens for antigen presentation in context of major histocompatibility complex I (MHC-I) and MHC-II and the induction of expression of chemokine receptors, pro-inflammatory cytokines, and costimulatory molecules. This activation program endows DCs with the capacity to traffic, via tissue-draining lymphatics, to T cell zones of secondary lymphoid organs to efficiently prime and control effector T cell responses (9).

**Figure 1:**
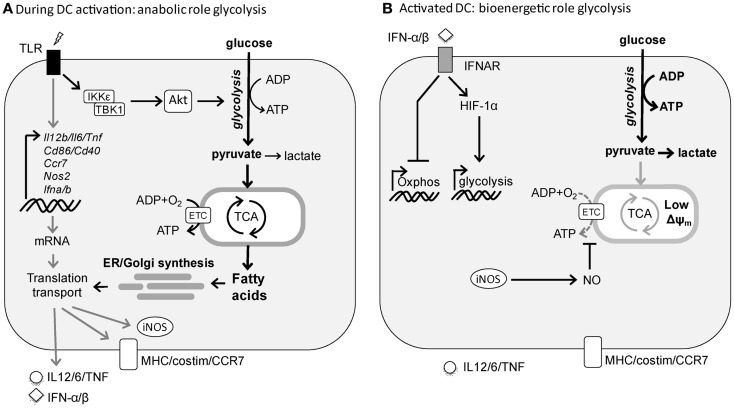
**TLR-induced metabolic changes in dendritic cells**. **(A)** Rapid induction of glycolysis in DCs by TLR stimulation serves an anabolic role in DC activation, by generating lipids for synthesis of additional membranes including ER and Golgi to support the increased demands of synthesis and transport of proteins required for DC maturation. **(B)** Following activation, DCs sustain high glycolytic rates to generate ATP to compensate for the loss of mitochondrial function. In cDCs this process appears to be driven by autocrine type I interferon, while in inflammatory DCs this is a direct consequence of iNOS-derived NO that blocks OXPHOS.

In T cells, catabolic metabolism centered around mitochondrial oxidative phosphorylation (OXPHOS) is associated with cellular longevity and quiescence, whereas cellular activation and proliferation are accompanied by a switch to glycolytic metabolism to support anabolic pathways needed for biosynthesis (4–6). Consistent with these observations, DCs when activated by TLR agonists, undergo a robust metabolic switch characterized by an increase in glycolysis and a concomitant progressive loss of OXPHOS (10–13). We have shown that in inflammatory DC subsets, such as murine GM-CSF-derived bone marrow DCs (14), this switch from OXPHOS to glycolysis is a direct consequence of TLR-induced inducible nitric oxide synthase (iNOS) expression that through the production of nitric oxide (NO) poisons the mitochondrial respiratory chain in an autocrine fashion (15). In this setting, in the absence of functional OXPHOS, TLR-agonist activated inflammatory DCs depend heavily on glycolysis as their sole source of ATP for survival both *in vitro* and *in vivo* (12). Consistent with this, *in vitro* and *ex vivo* TLR-activated *Nos2*^−/−^ inflammatory DCs still have functional OXPHOS and as result do not display a long-term increase in glycolytic metabolism (12). Likewise, we did not observe a switch to glycolytic metabolism following TLR stimulation of conventional DCs (cDCs) *ex vivo* (12), which do not express iNOS in response to TLR stimulation. However, a more recent *in vivo* study showed that TLR-activated cDCs do display long-term diminished mitochondrial activity and enhanced glycolysis (13). They found that this metabolic shift is iNOS-independent and instead driven by TLR-induced autocrine type I interferon production. Despite the differences in mechanism underlying the metabolic switch, similar to inflammatory DCs, cDCs seem to rely on the glycolytic shift for ATP production for their survival (13) (Figure 1).

These studies suggest that the metabolic reprograming toward glycolytic metabolism is a consequence of TLR-driven DC activation, rather than a prerequisite for it. However, given the fact that TLR stimulation results in a rapid activation program in both cDCs and inflammatory DCs, we recently tested the hypothesis that rapid metabolic reprograming needs to occur in both types of DCs to meet the bioenergetic and anabolic needs of TLR-driven DC activation itself. Indeed, we observed that TLR stimulation in both cDCs and inflammatory DCs results within minutes in an increase in glycolytic rate that is maintained for several hours after which it returns to prestimulation levels in the absence of iNOS (16). Inhibition of this early metabolic reprograming blunts DC activation, migration, and T cell priming both *in vitro* and *in vivo*, illustrating its importance for DC biology. Functionally, as opposed to the long-term glycolytic commitment, the rapid increase in glycolysis appears not to be important as a rapid source of ATP, but rather to serve a central anabolic role by acting as a carbon source for both the pentose phosphate pathway (PPP) and the tricarboxylic (TCA) cycle to support the generation of NADPH and citrate, respectively, that are used for *de novo* fatty acid (FA) synthesis. Moreover, glycolysis-supported *de novo* FA synthesis plays a crucial role in DC activation and function at the posttranscriptional level, by allowing for the synthesis and expansion of membranes including Golgi and ER that are required for synthesis, transport, and secretion of proteins associated with TLR-driven DC activation (16). These findings share strong parallels with activated T cells that heavily rely on glycolysis as a carbon source for *de novo* FA synthesis to support the need for membrane synthesis required for cellular proliferation (17). However, in contrast to T cells, DCs do not proliferate and seem to use this pathway to expand the cellular machinery necessary for increased production and secretion of the mediators that are integral to DC activation (Figure 1). This is consistent with a recent study positively correlating lipid content with immunogenicity of DCs in the liver and showing that the immunogenicity of DCs with high lipid content is dependent on FA synthesis (18). Taken together, these studies illustrate that the induction of glycolysis plays a central role for DCs to acquire immunogenic properties as well as their survival following activation.

While the metabolic features of immunogenic DCs are becoming more well characterized, there is still little known about the metabolism of tolerogenic DCs. Tolerogenic DCs, as opposed to immunogenic DCs, are generally characterized by the absence of traditional signs of activation, are maturation-resistant, and express increased levels of immunoregulatory factors, important for controlling Treg responses (19–21). Consistent with this immature, maturation-resistant phenotype, proteomic analysis of human DCs treated with dexamethasone and vitamin-D3, two well known immunosuppressive drugs that induce tolerogenic DCs, revealed increased expression of genes associated with mitochondrial metabolism and OXPHOS (22, 23). Furthermore, DCs conditioned by IL-4 acquire a phenotype highly reminiscent of alternatively activated (M2) macrophages and expression of M2-associated activation markers on DCs is required for optimal induction of IL-10-secreting T cells (24). The fact that M2 activation by IL-4 is dependent on increased fatty acid oxidation (FAO) and OXPHOS (25–27) makes it conceivable that there is a causal link between mitochondrial metabolism fueled by FAO and the acquisition of a tolerogenic phenotype by DCs. The observations that direct inhibition of glycolysis in TLR-activated DCs favors the induction of Foxp3-expressing Th cells at the expense of IFN-γ-producing Th1 cells (16), and that resveratrol and rosiglitazone, drugs known to promote FAO (28) and mitochondrial biogenesis (29), respectively, interfere with TLR-induced DC activation and can render them tolerogenic (30–33), would support this idea. However, these studies are mostly correlative and more work will be needed to elucidate whether there is a direct functional link between mitochondrial catabolic metabolism and the acquisition of tolerogenic properties of DCs.

## Regulators of DC Metabolism

In recent years, major advances have been made in unraveling the signaling pathways in immune cells that regulate their metabolic state. The conserved kinase mammalian/mechanistic target of rapamycin (mTOR) and its upstream activators PI3K-Akt have been identified as central regulators of cellular activation and proliferation due to their ability to control glycolysis and anabolic metabolism (34–36). Consistent with a role for mTOR in regulating DC metabolism as well, cDCs isolated from mice with a DC-specific deletion of tuberous sclerosis 1 (Tsc1), a negative regulator of mTOR, display enhanced mTOR activity, an increase of expression of glycolytic and lipogenic genes, and of maturation markers at steady state (37). Also in response to TLR ligands inflammatory DCs depend on signaling through PI3K, Akt, and mTOR for their long-term commitment to glycolysis (10, 38). mTOR promotes anabolic pathways and glycolysis by driving expression and stabilization of transcription factors such as sterol-regulatory element binding protein (SREBP) (39, 40) and hypoxia-inducible factor (HIF)-1α (41), that control expression of genes involved in lipogenesis and glycolysis, respectively. While it remains unknown whether SREBP plays a role in DC metabolism, several studies have documented an important role for HIF-1α in supporting the long-term commitment in glycolytic metabolism of both inflammatory DCs and cDCs in response to TLR stimulation (11, 13). Moreover, consistent with its well-recognized role in regulating innate immune cell function under inflammatory conditions (42), TLR-induced DC activation and T cell priming appear to rely on HIF-1α (11, 13, 43). However, whether this is a consequence of the role of HIF-1α in promoting glycolytic metabolism and thereby cell survival, or a reflection of the direct control of expression of inflammatory cytokines independently from glycolytic regulation (44, 45), remains to be addressed. In addition to direct transcriptional regulation of glycolysis through HIF-1α, mTOR may regulate the TLR-induced commitment to glycolysis indirectly in inflammatory DCs, through induction of iNOS expression and NO production (46, 47) that forces these cells to switch to glycolysis in the absence of mitochondrial respiration (12).

In contrast to the clear role for the mTOR-HIF-1α axis in regulating TLR-induced long-term metabolic changes, recent evidence suggests that the early TLR-driven induction of glycolysis to support the anabolic demands of DC activation itself does not depend on mTOR or HIF-1α signaling (16, 48). Instead, there is a critical role for Akt in this response that directly enhances the enzymatic activity of rate-limiting glycolytic enzyme hexokinase-II (HK-II) by promoting its association with the mitochondria. Interestingly, Tank-Binding Kinase 1 (TBK1) and IκB kinase-(IKK) but not the canonical Akt activators PI3K or mTORC2 appear to be the crucial upstream regulators of Akt activation in this TLR-driven rapid induction of glycolysis (16). Taken together based on these recent findings a picture is emerging that TLR-signaling drives two functionally and temporally distinct waves in glycolytic metabolism in DCs that are controlled by largely separate signaling pathways (Figure 1).

**Figure 2:**
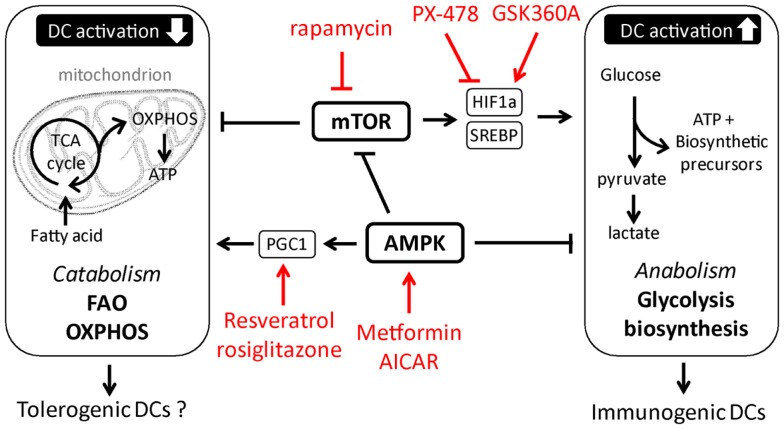
**Putative metabolic pathways and upstream regulators in tolerogenic versus immunogenic dendritic cells**. In red examples are depicted of pharmacological approaches currently tested or used in other therapeutic settings, that could be used to manipulate DC metabolism.

While the signaling pathways that promote the shift to glycolysis and anabolic metabolism required for TLR-induced activation and immunogenicity of DCs are starting to be characterized, much less is known about the signals in DCs that may antagonize these responses and that are potentially important for induction and function of tolerogenic DCs. In this respect, in T cells and in macrophages the metabolic sensor AMP Kinase (AMPK) is known to play a central role in antagonizing biosynthetic pathways, including lipogenesis, and has instead been shown to promote catabolic metabolism by, amongst other pathways, the activation of peroxisome proliferator-activated receptor gamma coactivator (PGC)-1α that promotes mitochondrial biogenesis to increase mitochondrial OXPHOS (7, 35). Consistent with these observations, pharmacological activation of AMPK suppresses TLR-induced glucose consumption and activation of DCs, while knockdown of AMPK has the opposite effect (10, 49), suggesting an important role for AMPK signaling in the metabolic control of DC activation. Furthermore, systemic administration of drugs activating AMPK signaling to promote catabolic metabolism drives induction of tolerogenic immune responses in several inflammatory disease models (50–52). However, it remains to be determined whether these treatments exert their effects through direct induction of tolerogenic DCs. Moreover, resveratrol, a drug that has been linked to induction of tolerogenic DCs, is thought to favor catabolic metabolism through activation of the histone deacetylase Sirtuin 1, which is known to suppress HIF-1α function as well as enhance PGC-1α activity (29, 32, 53). In addition, DCs deficient for Nuclear factor-erythroid 2 p45-related factor-2 (NRF2) or PPAR-γ, downstream targets of PGC-1α, display increased maturation and T cell priming capacity (31, 54, 55). Hence, these studies may point toward an important role for the AMPK-PGC-1α axis in promoting mitochondria-centered catabolic metabolism in DCs, which may be crucial for the acquisition of a tolerogenic phenotype (Figure 2). However, how these signaling pathways are regulated under physiological conditions and to what extent the effects of these factors on DC biology can be attributed to direct regulation of DC metabolism are still unresolved questions.

## Manipulating DC Metabolism for Therapeutic Purposes?

There is a great interest in the use of DCs as targets for immune-intervention and for vaccine strategies, because of their powerful immune stimulatory as well as regulatory functions (56). The use of highly immunogenic DCs can be used to promote robust cellular and humoral immunity that is central for improving vaccination efficacy against a variety of infectious diseases and tumors, while the use of tolerogenic DCs will allow for induction of regulatory immune responses in settings where unwanted effector T cell responses need to be controlled, such as to prevent rejection following transplantation. It is of pivotal importance to identify and characterize the regulatory processes underpinning these different functions of DCs. It is becoming clear from the aforementioned studies that the activation and T cell-priming function of DCs is tightly regulated by their metabolic fate. What can we learn from these new metabolic insights in DC biology and would there be ways to use this knowledge in developing approaches to enhance DC-based immunotherapies? The idea of manipulating cellular metabolism for therapeutic purposes is not a new concept. In fact, in the cancer field there is great interest in the use of pharmacologicals that inhibit anabolic metabolism or glycolysis to reduce tumor growth (57–60). Likewise, studies in T cells have provided a clear proof of principle that targeting of cellular metabolism can provide a viable means for improving the efficacy of vaccinations (61, 62).

Based on the importance of anabolic metabolism and glycolysis in supporting DC activation and immunogenicity, and the possible role of catabolic metabolism in supporting tolerogenic DC function, it will be of great interest to assess whether promoting these types of metabolism in DCs can be used as a strategy to enhance the immunogenicity or tolerogenicity of DCs in therapeutic settings. It should be noted that some of the pharmacological approaches currently used to manipulate the immunogenicity of DCs, such as dexamethasone, Vitamin-D3, and rapamycin (63–66) that are known for their capacity to induce tolerogenic DCs, have been described to influence DC metabolism (22, 23, 38). Thus it is possible that direct targeting of metabolism of DCs as a single treatment may not be superior to some other already existing manipulations that also affect metabolism. It is therefore more conceivable that manipulation of metabolism of DCs for immunotherapy will be most effective when used in conjunction with existing approaches to complement and enhance their therapeutic efficacy. A second important advantage of direct enforcement of certain types of metabolism in DCs is that is it may render them more resistant to environmental metabolic manipulation. This is highly relevant since a key parameter that determines the efficacy of immunotherapies is how long targeted DCs retain their phenotype following their functional manipulation. The microenvironment, which DCs become exposed to *in situ*, may lead to the loss of immunogenicity or tolerogenicity and would significantly affect the outcome of the therapy. For instance, the immunostimulatory capacity of DCs is often suppressed in a tumor microenvironment (67). Given the important role for cellular metabolism in regulating DC function, many of the suppressive effects of tumors appear to be attributable to effects on DC metabolism. It has been shown that tumor-derived IL-10 can suppress glycolysis in DCs through down regulation of glycolytic enzyme pyruvate kinase (68). Additionally, yet unidentified tumor-derived factors can promote aberrant lipid accumulation in DCs, resulting in impaired T cell priming (69, 70). Moreover, immunogenic DCs are likely to be impaired in their function in a microenvironment where glucose will be scarce due to the high glycolytic rates of tumors themselves (71). Finally, caloric intake and mitochondrial activity are important determinants of organismal as well as cellular lifespan (72, 73). Therefore targeting DCs metabolism can also be used to manipulate DC longevity to affect their immunostimulatory potential. For example, mTOR inhibition has shown to increase the lifespan of TLR-activated DCs and enhance their capacity to induce protective tumor immunity (38).

Several agonist and antagonists of metabolic enzymes and upstream signaling pathways that could be used to manipulate DC metabolism have already been developed and tested for safety and efficacy in other systems (58–60, 74) (Figure 2). In addition to pharmacological approaches, genetic manipulation through introduction of small hairpin RNAs has shown to be a successful strategy to alter DC immunogenicity (75, 76) and could provide a feasible alternative to target DC metabolism. In recent years, there has been a major focus on manipulating the immunostimulatory properties *ex vivo* generated DCs for autologous DC vaccination. Some of these vaccines have made it to the clinic (77) or are currently in clinical trials (78–80). In addition, widespread enthusiasm has been generated by results from the *in vivo* use of nanoparticles, consisting of antibody covered micelles carrying antigens and potentially drugs or shRNA, that can be specifically targeted to DCs *in situ* (81, 82). Given the amenability of cellular metabolic intervention, it seems feasible that metabolism-targeted manipulations to DCs could be implemented in protocols for DC-based vaccinations.

## Concluding Remarks

It is becoming increasingly clear that the metabolic phenotype of DCs dictates their activation and immunogenicity. However, many of the details and underlying mechanisms of how cellular metabolism controls the functional properties of DCs remain to be determined. For instance, the precise metabolic processes that underpin the function of tolerogenic DCs are still poorly defined. Moreover, do different *in vivo* DC subsets have different metabolic characteristics and are unique metabolic processes required for DCs to perform particular functions, such as cross presentation or the induction of Th1/2/17 cell responses? Addressing these and other questions will not only contribute to a better fundamental understanding of the biology of DCs, but will also aid in the rational design of metabolism-based approaches to enhance the efficacy of DC-based immunotherapies.

## Conflict of Interest Statement

The authors declare that the research was conducted in the absence of any commercial or financial relationships that could be construed as a potential conflict of interest.
